# Antimicrobial Consumption and Resistance in a Tertiary Care Hospital in Jordan: Results of an Internet-Based Global Point Prevalence Survey

**DOI:** 10.3390/antibiotics9090598

**Published:** 2020-09-13

**Authors:** Khawla Abu Hammour, Esraa AL-Heyari, Aya Allan, Ann Versporten, Herman Goossens, Ghayda’ Abu Hammour, Qusai Manaseer

**Affiliations:** 1 Department of Biopharmaceutics and Clinical Pharmacy, Faculty of Pharmacy, The University of Jordan, Amman 11942, Jordan; 2 Clinical Pharmacist Pharmacy Department—Jordan University Hospital, The University of Jordan, Amman 11942, Jordan; esraa_heyari92@yahoo.com (E.A.-H.); ayaallan92@yahoo.com (A.A.); gabuhammour@gmail.com (G.A.H.); 3 Laboratory of Medical Microbiology, Vaccine & Infectious Disease Institute, University of Antwerp, 2000 Antwerpen, Belgium; ann.versporten@uantwerpen.be (A.V.); Herman.Goossens@uza.be (H.G.); 4 Faculty of Medicine, The University of Jordan, Amman 11942, Jordan; qusaiw100@live.co.uk

**Keywords:** antimicrobial agents, stewardship program, resistance

## Abstract

**Background:** The Global Point Prevalence Survey (Global-PPS) provides a standardised method to conduct surveillance of antimicrobial prescribing and resistance at hospital level. The aim of the present study was to assess antimicrobial consumption and resistance in a Jordan teaching hospital as part of the Global-PPS network. **Methods:** Detailed antimicrobial prescription data were collected according to the Global Point Prevalence Survey protocol. The internet-based survey included all inpatients present at 8:00 am on a specific day in June–July 2018. Resistance data were based on microbiological results available on the day of the PPS. **Results:** Data were collected for 380 patients admitted to adult wards, 72 admitted children, and 36 admitted neonates. The overall prevalence of antimicrobial use in adult, paediatric, and neonatal wards was 45.3%, 30.6%, and 22.2% respectively. Overall, 36 patients (7.4%) were treated for at least one healthcare-associated infection (HAI). The most frequent reason for antimicrobial treatment was pneumonia. Cephalosporins and carbapenems were most frequent prescribed among adult (50.6%) and paediatric/neonatal wards (39.6%). Overall resistance rates among patients treated for a community or healthcare-associated infection was high (26.0%). Analysis of antibiotic quality indicators by activity revealed good adherence to treatment guidelines but poor documentation of the reason for prescription and a stop/review date in the notes. **Conclusion:** The present study has established baseline data in a teaching hospital regarding the quantity and quality of prescribed antibiotics in the hospital. The study should encourage the establishment of tailor-made antimicrobial stewardship interventions and support educational programs to enhance appropriate antibiotic prescribing.

**What Is Already Known**

- Inappropriate use of antimicrobials in hospitals is widely reported.
- In Jordan, understanding of antimicrobial consumption patterns is required to support recent policy intentions.

**Added Value:**

- Our results add to WHO’s recent efforts to understand the prevalence of infections and antibiotic consumption in low- and middle-income countries.
- Evaluation of both the quality and quantity of prescribed antimicrobial agents may enhance the improvement of their prescribing patterns. This could be achieved through positively changing the education and prescribing practices, mainly in countries that lack appropriate tools, including ours, to monitor antibiotic prescribing in hospitals.
- The results of the Global Point Prevalence Survey will allow sharing of best practice, raise awareness of inappropriate antimicrobial prescribing to improve the prescribing of antibiotic at hospital level.
- Governments can utilise this data to provide a base for antimicrobial stewardship programs.

## 1. Introduction

Inappropriate utilisation of antibiotics is an important and changeable driver for antimicrobial resistance (AMR), linked with higher rates of mortality and morbidity [1] in addition to the extra unjustified cost [2,3]. AMR is of particular concern in low- to-middle income countries, including Jordan, as a result of the recent increase in the use of marketed antimicrobial agents inappropriately [4,5,6,7,8], this rising in the rates of AMR has enhanced the development of national and international initiatives to improve the use of antibiotics in the future by implementing programs of antimicrobial stewardship [9,10,11].

One of the important barriers to establish and implement a successful stewardship program worldwide is the scarcity of data regarding the quality and quantity of antimicrobial use [12]. Therefore, improvement of assessment and decision making of interventions need the development of surveillance systems to monitor antimicrobial use in the hospital settings [13]. The main aim of the Global Point Prevalence Survey (PPS) of Antimicrobial Consumption and Resistance is to identify the international prevalence of the antimicrobial use, with an emphasis on countries with low support, resources, and expertise. This method of a PPS was used in the present study during 2018 to report the antibiotic prescribing practices for inpatients admitted to hospital wards. The variation in the quantity of antibiotic use was assessed across these wards in a teaching hospital. Next, the quality of antibiotic use was assessed by a set of antibiotic quality indicators, which can help in identifying targets for intervention and improvement.

## 2. Methods

### 2.1. Study Design, Setting, and Ethics

A cross-sectional audit of antimicrobial prescribing practices and the presence of antimicrobial resistance was conducted in in June–July 2018 at a tertiary teaching hospital in Jordan; an upper-middle-income country in the Middle East Jordan University Hospital, the first academic teaching hospital in the Hashemite Kingdom of Jordan with 600 beds. It encompasses all major and submedical and clinical specialties comprising 64 different specialties. It also conducts about twenty thousand surgical operations annually, in addition to treating more than half a million patients a year. The standardised Global Point Prevalence Survey method was used to gather detailed data regarding the prescription of antimicrobial agent and their resistance type (www.global-pps.com). Data were collected for patients admitted to all hospital wards. The internet-based survey included all inpatients (adults, children, and neonates) present at the ward on a specific day (denominator) in June–July 2018. Detailed patient and antimicrobial information was collected for patients who received an antimicrobial agent at 8:00 am on the day of the PPS (numerator).

Ethical approval for the present study was obtained from the hospital institutional review board (10/2018/11111).

### 2.2. Data Collection

Hospital-based clinical pharmacists were responsible for completing the Global-PPS. Two responsible pharmacists did a one-day survey, during which all wards had to be audited once. All inpatients who were at the ward at 8:00 a.m. were included (denominator). Two forms were completed by the clinical pharmacists. Ward-level data, such as the total number of inpatients an the ward, were documented in the first form, and the second form was for patient-level data. For each patient who was prescribed and received at least one antimicrobial agent at 8:00 a.m. on the day of the PPS, we gathered data including baseline patient characteristics, the prescribed antimicrobials, their diagnosis according to a predefined list, and whether it concerned treatment for a community-acquired (CAI) or healthcare-associated infection (HAI) or prophylactic prescribing (for both medical or surgical prophylaxis). For surgical prophylaxis, the administration had to be assessed in the previous day to enable surgical prophylactic prescribing either as one single dose, one day, or more than one day. The full protocol can be found in the Supplemental Material.

Quality indicators of prescribing the antimicrobial agents included the guideline compliance that refers to antibiotic choice (not route, dose, duration) in compliance with local guidelines, recording of a stop or review date for the antimicrobial in the medical files, and the documentation of the diagnosis at the beginning of the treatment/prophylaxis in the medical files of patients. Moreover, next to empirical treatment, we recorded also if it concerned targeted treatment that is based on the results of microbiology data from a relevant clinical specimen, such as blood or sputum. An online standardised and internationally recognised WHO anatomical therapeutic chemical classification system (ATC) (2017 version) [14,15] was used to automatically classified the antimicrobial agents in the present study including the antibacterials, antifungals and antivirals for systemic use as well as antibiotics used to treat tuberculosis.

Discussion and personal judgment during the survey regarding the appropriateness of prescribing of antimicrobial agents were not allowed. All data were entered into the freely available Global-PPS program, an internet-based application for anonymised data entry.

### 2.3. Data Analysis

The rates of antimicrobial prescribing were presented as a percentage of patients on antimicrobials according to the formula 100 × (number of treated patients/total number of admitted patients at 8:00 a.m. on the day of the survey), or as a percentage of antibiotic or antimicrobial prescriptions out of all antibiotic/antimicrobial prescriptions (proportional use).

Resistance rates are provided for the hospital overall as well as by resistance type. These are calculated as the number of patients which received a microbiology-based treatment available on the day of the PPS for a certain resistance type/total number of patients receiving a treatment with at least one antibacterial for systemic use (ATC J01) for a community-acquired or healthcare-associated infection.

## 3. Results

Data were collected for 380 patients admitted to adult wards, 72 admitted children, and 36 admitted neonates in the hospital.

### 3.1. Prevalence of Prescribing Antimicrobial Agents

Out of 380 admitted adult patients, 172 (45.3%) received at least one antimicrobial (41.5%, 43.0%, and 82.8% in the adult medical, surgical, and intensive care units, respectively) and out of 72 admitted children, 22 (30.6%) received at least one antimicrobial on the day of the PPS (30.0%, 25.0%, and 50.0% in paediatric medical, surgical, and intensive care units, respectively). Out of 36 neonates, 8 (22.2%) received at least one antimicrobial on the day of the PPS; of which all were admitted on a neonatal intensive care unit (8/18 neonates, 44.4%).

### 3.2. Prevalence of Healthcare-Associated Infections

Out of 488 admitted patients, 36 patients (7.4%) were treated for at least one HAI, including 7.9% (n = 30/380 patients) in adult wards and 5.6% (n = 6/108 patients) in paediatric and neonatal wards).

### 3.3. Indications for Antimicrobial Prescribing

Pneumonia or lower respiratory tract infections (22.4%) were the most common indication, followed by the skin and soft tissue infections (14.4%) (Table 1).

This implies that a patient with multiple diagnoses can be counted several times. Neonates admitted on neonatal intensive care units or neonatal medical wards, prophylactic prescribing and prescribing for unknown reasons are excluded.

### 3.4. Prescription Patterns of Antimicrobial Agents

Out of 309 antimicrobial prescriptions, 258 were prescribed in adult wards. Overall, antibacterials for systemic use (J01) represented 92.2% (n = 285) of all prescriptions, followed by antimycotics for systemic use (J02, 3.9%, n = 12), nitroimidazole derivatives (P01AB, 1.3%, n = 4), antibiotics prescribed as intestinal anti-infectives (A07AA, 1.0%, n = 3), antivirals (J05, 1.0%, n = 3), and drugs to treat tuberculosis (J04A, 0.6%, n = 2).

Overall, the most frequently prescribed antibiotics were the other beta-lactam antibacterials (ATC J01D, cephalosporins and carbapenems) (50.6% (n = 120) and 39.6% (n = 19) of prescriptions among adult and paediatric/neonatal wards, respectively). Other antibacterials (ATC J01X) including glycopeptide antibacterials, polymyxins, steroid antibacterials, imidazole derivatives, nitrofuran derivatives were the second most prescribed antibiotics (22.4% (n = 53) and 22.9% (n = 11) of prescriptions among adult and paediatric/neonatal wards, respectively; mainly parenteral vancomycin). Beta-lactam antibacterials (ATC J01C, penicillins) were the third most frequently prescribed antibiotics (10.5% (n = 25) and 20.8% (n = 10) of prescriptions among adult and paediatric/neonatal wards, respectively, mainly piperacillin and tazobactam in adult wards and ampicillin in paediatric/neonatal wards). The overall proportional antibiotic use of antibacterial agents for medical patients, surgery patients, adult ICU patients, paediatric, and neonatal ICU patients are portrayed in Table 2.

Most therapeutic antimicrobial agents were used for community-acquired infections (69.8%) (n = 141/202), of which 85.8% (n = 121/141) were for empiric versus 14.2% (n = 20/141) for targeted treatment, whereas less than one-third (30.2%) (n = 61/202) of therapeutic antimicrobial agents were used to treat healthcare-associated infections, of which 62.3% (n = 38/61) were for empiric versus 37.7% (n = 23/61) for targeted treatment.

During this survey, results revealed that the majority of prophylactic antimicrobial use (80.0%, n = 12/60) was for surgical prophylaxis compared to only one-fifth (20%, n = 48/60) for medical prophylaxis. These antimicrobials include the antibacterials, antifungals, and antivirals for systemic use. Medical prophylaxis encompasses a wide range of antimicrobials which are prescribed for medical prophylactic reasons. It refers for example to long-term prescribing to prevent urinary tract infections or the use of antifungals in oncology patients.

Assessment of the antibiotics that were used for sepsis (treated patients = 2) revealed that the top four most frequently used in adults and children were vancomycin, piperacillin and tazobactam, levofloxacin, and imipenem with cilastatin (25% each). On the other hand, the most frequently used antibiotics for pneumonia in adults and children (treated patients = 28) were piperacillin and tazobactam (28.0%), imipenem and cilastatin (18.0%), ceftriaxone (18.0%), meropenem (13.0%), azithromycin (12.0%), and vancomycin (11.0%).

For medical prophylaxis in adults and children (n = 9 patients), the top three most frequently used antimicrobials were fluconazole (42.0%, n = 5 prescriptions), azithromycin, and imipenem and cilastatin (18.0%, both 2 prescriptions), while for surgical prophylaxis (n = 41 patients), it was found that ceftriaxone (39.6%, n = 19 prescriptions), cefuroxime (16.7%, n = 8 prescriptions), and cefazolin (10.4%, n = 5 prescriptions) were most commonly prescribed among adults and children.

Results indicated that for surgical prophylaxis of the gastrointestinal tract in adults and children (n = 5), the most commonly prescribed antimicrobial agent was cefuroxime (66.0%, n = 4 prescriptions). Prescribed antibiotics for surgical prophylaxis for urinary tract procedures in adults and children (= 6) included imipenem and cilastatin (50.0%, n = 3 prescriptions), followed by ceftriaxone (32.0%, n = 2) and ertapenem (18.0%, n = 1).

### 3.5. Quality Indicators and Prescription Patterns of Antimicrobial Agents

**Figure 1:**
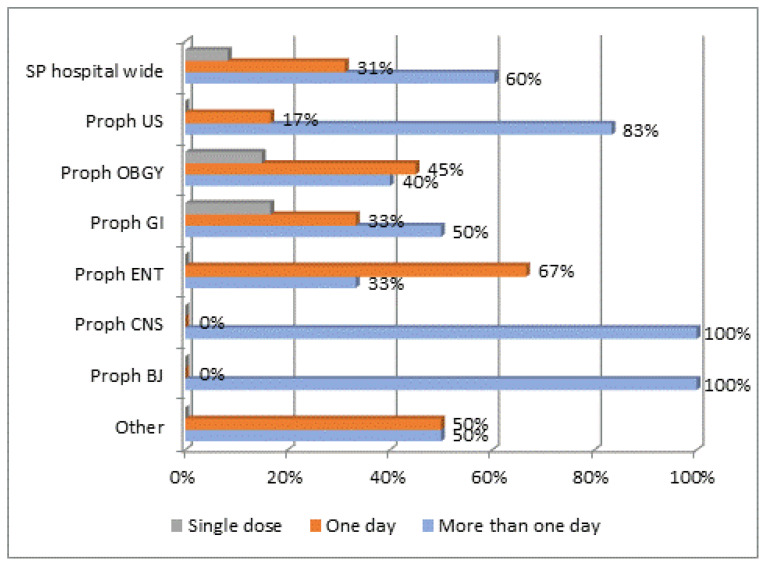
Duration of antimicrobial surgical prophylaxis (SP) in adults (N = 36) and children (N = 5) according to the type of surgical procedure.

Several antimicrobial quality indicators were assessed during the present survey. The first quality indicator was in regard to the duration of surgical prophylactic antibiotics. A total of 41 patients (adult and children) were prescribed 46 antibiotics for surgical prophylaxis, of which 8.7% were prescribed as a single dose, 30.4% for more than one dose for one day, and 60.9% for more than one day. This duration varied according to the surgery as presented in Figure 1.

Proph US = Prophylaxis for urological surgery (including 2 children); OBGY = obstetric or gynaecological surgery; GI = gastrointestinal tract; ENT = ear, nose, throat (including 2 children); CNS = central nervous system; BJ = plastic or orthopaedic surgery (bone or joint) (including 1 child).

Guideline compliance was lowest for surgical patients (66.0%, 64/97 prescriptions), of which mainly ceftriaxone was not prescribed according to the guidelines for various indications. In addition, the documentation of a stop or review date of the antibiotic prescription was low for all included wards in the hospital. The majority of patients in medical, surgical, or ICU received intravenous antibiotics (Table 3).

The next quality indicator investigated how frequent the antimicrobial prescription was based on microbiology data. Targeted prescribing accounted for 21.5% of all antimicrobial prescriptions for therapeutic use (CAI and HAI) (Table 3). Remarkably, 25.5% of all prescriptions for surgical indications were targeted, of which 84.6% (11/13 prescriptions, n = 10 patients) were targeting bone and joint infections.

### 3.6. Resistance Patterns

Table 4 provides an overview of identified multidrug resistant organisms for a total of 33 patients resulting in an overall resistance prevalence of 26.0%. MRSA (7.1%) and other identified multidrug resistant organisms (MDRO) (8.7%) that were most often isolated. Other MDRO were those identified not belonging to the 8 MDRO under surveillance.

## 4. Discussion

The Global-PPS provides a simple technique to focus on antibiotic prescribing and resistance on an international scale. In the hospital, the clinical pharmacy team assessed (for the first time) the prescribing patterns of antibiotics and collected information regarding their resistance using the Global-PPS. These data are crucial to develop the antimicrobial stewardship programs.

A substantial difference between and within the included wards in the present study were identified with the highest level of antimicrobial prescribing in adult intensive care units (82.8%) and the lowest in medical wards (41.5%). The overall prevalence for all patients (45.3%) was similar to that in a survey done in 183 US hospitals in 2011 (49.9 [95% CI 49.0–50.9) [16] and higher than the weighted prevalence of previous European surveys in 2011–2012 (32.6%) [17]. On the other hand, the overall prevalence of HAI in the hospital of 7.4% was comparable to previous 2015 Global-PPS results observed among Southern European countries (7.5%) but lower than that of the Asian and American regions (range 8.7%–11.9%) [18].

A closer examination of the indications for antibiotic prescription revealed that pneumonia or lower respiratory tract infection, skin, and soft tissue followed by urinary tract infections were the most frequent indications for antimicrobial agents in the present study. This is comparable to what has been reported in a previous global point prevalence survey in 53 countries [18]. Βeta-lactams other than penicillin were the most frequently prescribed antibiotics in the present study, with a high frequency of prescription in surgery patients. Penicillin was the most frequently prescribed antibiotic in neonatal ICU patients. Additionally, it was noted that there was the frequent use of levofloxacin in hospitals in Jordan mainly for both types of pneumonia. This is comparable to what has been reported in Asia (7.4% in the east and south Asia compared to 0.9% in the West and Central Asia [18]. Differences in cost of or access to fluoroquinolones could explain their prescription patterns in different countries. Moreover, marketing strategies and antibiotic regulations are playing a major role in the use of these antibiotics [19].

Another remarkable note was that the most frequently used antibiotics for pneumonia in adults and children were the broad-spectrum antibiotics. These include vancomycin, meropenem, azithromycin, imipenem with cilastatin, ceftriaxone, and piperacillin and tazobactam in addition to high-frequency use of vancomycin in different wards in the hospital. This could be due to a high reported prevalence of methicillin-resistant *Staphylococcus aureus* (MRSA) infections which is in line with the high proportion of patients who received targeted treatment against MRSA infections (7.1%) in our survey. Reports in Jordan suggest high carriage rates of MRSA (7.5–19%) among healthy individuals [20]. Moreover, an important note was that high number of last-line antibiotics have been used for urinary tract infections (UTIs) to treat Gram-negative bacteria. Additionally, azithromycin is often used in our hospital due to its demonstrated efficacy and safety in reducing respiratory complications in patients who are suffering from respiratory diseases [21].

Several multidrug resistant organisms were identified for a total of 33 patients out of 127 patients which were treated for a CAI or HAI. This resulted in an high overall resistance prevalence of 26.0% as compared with the mean resistance prevalence of European countries participating in the Global-PPS in 2015 (6.8%; 25 countries, 213 hospitals; unpublished Global-PPS results, but available for participants through the online individual feedback reports) or, for example, Canada where an overall resistance prevalence of 8.5% was found in 14 Canadian hospitals in 2017 [22]. Prevalence rates for the different resistance types identified in the hospital, such as MRSA (7.1%) and other MDRO (8.7%) are indeed all higher as compared to mean results for the different European regions [18]. These isolated multidrug resistant organisms could be reduced by infection prevention control measures and tailor-made antimicrobial stewardship interventions. Six antimicrobial quality indicators were assessed to identify inappropriate prescribing patterns of antimicrobial agents in the present survey. They could help to set benchmarks for quality improvement of antimicrobial use in hospitals. The documentation of the reason of the antimicrobial prescription was absent in almost half of the prescriptions for medical and surgical indications and in 10% of the ICU wards. The ICU’s findings (documentation) were encouraging and mirror findings of the quality indicator (80%) in the European Surveillance of Antimicrobial Consumption point prevalence survey which was conducted in 2009 among European adults [17]. The findings for medical and surgical indications were, however, lower than that of the African countries in the Global-PPS study [18]. The documentation of the reason for prescription ensures communication of treatment based on the right diagnosis among physicians and other healthcare workers and allows recording of stopping or reviewing the antimicrobial prescription as well as other interventional processes such as de-escalation in the hospital. Results of the present study further revealed that a stop/review date was poorly documented (in less than 15% of prescribed antimicrobials). Consequently, these two indicators should be targeted as a key metrics for a stewardship program in the hospital.

Developing, updating, and educating on local empiric treatment guidelines would likely increase adherence to the guidelines which could lead to improvement in clinical outcomes such as duration of treatment, mortality, and length of hospitalisation. The present study revealed a satisfactory compliance with local empiric treatment guidelines in medical and ICU wards. However, a lower compliance was observed for surgical patients which needs further investigation in order to cut down the prescription of broad-spectrum antibiotics for surgical prophylaxis. However, the observed rates were similar to figures reported in previous surveys in Africa, Latin America, and Central and West Asia [18]. A systematic review and meta-analysis [23] revealed that guideline-adherent empirical therapy was linked with significant relative risk reduction for mortality of 35%. The reasons for poor compliance with guidelines remain uncertain and could be due to several factors such as clinical uncertainty, local resistance patterns, and fear of treatment failure.

Similar to previous studies [24,25], a high proportion of broad-spectrum antibiotic prescribing for surgical prophylaxis was noticed for several types of surgeries for more than one day in the included wards. Prolonged surgical prophylaxis was also common in Southern and Eastern Europe (85%, 86.3% respectively) [17,18], although it does not prevent the development of postoperative infections. Instead, it may increase the risk of adverse events and antimicrobial resistance. In the absence of preoperative infections or serious complications, it is unnecessary to prescribe prophylactic antibiotics for more than one day [26,27,28]. Further research is warranted to clarify the reasons for this pattern, particularly in plastic and orthopaedic surgeries. Our results are similar to previous studies done in Jordan. Jalil et al. demonstrated the low level of overall compliance with surgical antimicrobial prophylaxis guidelines. The most frequent deviation from the guidelines was extended administration of prophylactic antibiotics (88.2%) of the study population. They recommended to give a central role to hospital pharmacists in managing the practice of surgical antibiotic prophylaxis [29,30].

The pharmacy department discussed these findings with the hospital management in 2019 to establish a multidisciplinary antimicrobial stewardship (AMS) team as part of an antimicrobial stewardship program. Internal educational AMS workshops have already been taken place in 2019 targeting physicians, pharmacists, and nurses. Questionnaires were distributed before and after these workshops to assess their knowledge on AMS. Furthermore, as an initial start, clinical pharmacists were assigned beginning 2020 to enhance appropriate surgical prophylactic antibiotic use for caesarean section interventions. Importantly, tailor-made interventions with target setting should further be developed and performed, after which the impact could be assessed with repeated PPSs.

### Strengths and Limitations

The strengths of the study include validation and the completeness of the data through the internet-based tool, as well as the opportunity for real-time educational feedback of the outcomes to the hospital. There was a minimal requirement for training, and we had successful participation in this global survey with support from the online materials and helpdesk support. On the other hand, the description of prescribing patterns in one hospital through the epidemiological methods of our cross-sectional survey is considered as one of the main limitations. This one-time snapshot measurement cannot be considered as representative for the hospital. Moreover, another limitation is the lack of previous data for this type of study. Thus, there was nothing to compare the results to.

Hopefully, in future global surveys, an increased number of Jordan hospitals could participate. This would provide a more meaningful and representative picture of antimicrobial prescribing and resistance at the country level.

## 5. Conclusions

This present study has established baseline data in a teaching hospital to establish and support educational programs regarding antimicrobial stewardship programs and ultimately improve the utilisation of antibiotics. Through this study, there was good support from experienced partners and good engagement from our clinical pharmacists. This is an important issue in countries with limited personnel and financial resources.

Several opportunities for improvement became obvious during the study, including developing local prescribing guidelines and adherence to these. Adherence to the WHO recommendations regarding surgical prophylaxis should take priority. Moreover, there is an urgent need to enhance the use of diagnostic tests to reliably support the selection of appropriate antibiotics. A follow-up process will be conducted as part of ongoing programs to support the antimicrobial stewardship program. Future participation in the Global PPS is also planned in response to the COVID-19 pandemic, with increased training for healthcare workers in order to avoid unnecessary antibiotic use and the implementation of strict infection prevention and control measures.

## Figures and Tables

**Table 1:** Most common indications for therapeutic antibiotic prescription (CAI and HAI) among adults and children.

Diagnosis *	Patients (n = 125)	%
Pneumonia or lower respiratory tract infection	28	22.4
Skin and soft tissue	18	14.4
Upper urinary tract infection	15	12.0
Bone joint infections	14	11.2
Lower urinary tract infection	11	8.8
Intraabdominal sepsis	6	4.8
Other	6	4.8
Gastrointestinal infections	5	4.0
Bacteraemia	4	3.2
Infection of central nervous system	4	3.2

* Count on the number of diagnoses treated with at least one antimicrobial.

**Table 2:** Overall proportional antibiotic use (ATC J01) for medical, surgery, adult ICU, paediatric ICU, and neonatal ICU patients.

Overall Proportional Antibiotic Use	Medical Patients (110 Prescriptions)	Surgery Patients (109 Prescriptions)	Adult ICU Patients (47 Prescriptions)	Paediatric ICU Patients (6 Prescriptions)	Neonatal ICU Patients (13 Prescriptions)
Other beta-lactams	46.4%	62.4%	34.0%	50.0%	7.7%
Other antibacterials	18.2%	23.9%	27.7%	33.0%	23.1%
Penicillins	14.5%	7.3%	12.8%		38.5%
Sulfonamides and Trimethoprim			2.1%		
Macrolides Lincosamides and Streptogramins	4.5%	3.7%	2.1%	16.7%	
Quinolones	11.8%		8.5%		
Tetracyclines		1.8%	6.4%		
Aminoglycosides	4.5%	0.9%	6.4%		30.8%

**Table 3:** Prevalence of key quality indicators for antibiotic prescribing (all patients, ATC code = J01; %).

Quality Indicator	Overall (197 Patients on 285 Antibiotics)	Medical (75 Patients on 110 Antibiotics)	Surgical (86 Patients on 109 Antibiotics)	ICU (36 Patients, 66 Antibiotics)
Indication recorded	180 (63.2%)	60 (54.5%)	61 (56.0%)	59 (89.4%)
Stop/review date documented	24 (8.4%)	8 (7.3%)	12 (11.0%)	4 (6.1%)
Guidelines missing	12 (4.2%)	5 (4.5%)	7 (6.4%)	0 (0%)
Guideline compliant	138/187 (73.8%)	55/65 (84.6%)	46/76 (60.5%)	37/46 (80.4%)
Intravenous route of administration	191 (97.0%)	71 (94.7%)	85 (98.8%)	35 (97.2%)
Multiple ATB diagnosis *	72/205 (35.1%)	28/78 (35.9%)	22/87 (25.3%)	22/40 (55.0%)
Multiple ATB patient **	71 (46.2%)	27 (36.0%)	23 (26.7%)	24 (66.7%)
Targeted prescribing ***	41/191 (21.5%)	16/90 (17.8%)	31/51 (25.5%)	12/50 (24.0%)

Five patients are not counted as they were not prescribed an antibacterial for systemic use but other antimicrobials. For indication recorded, and stop/review date documented: count at antibiotic (ATC code = J01) level. For where guidelines were missing: count on no guideline for an indication at patient level and diagnosis over total scores for this indicator. For guideline compliance: Count at patient level and diagnosis for compliance equals yes/yes plus no. Intravenous route of administration is calculated at patient level. For combination therapy with >1 antibiotic: if one antibiotic by diagnosis is not compliant, this combination therapy, as a whole, for this diagnosis will be counted as noncompliant. Intravenous route of administration calculated at patient level. * Multiple antibiotics (ATB) diagnosis is defined as receiving more than one antibiotic for a single identified reason to treat at patient level. ** Multiple ATB patient is defined as receiving more than one antibiotic at patient level. *** Targeted prescribing is defined as N antibiotics prescribed based on a microbiological result out of “all therapeutic prescribing for community and healthcare-associated infections” only.

**Table 4:** Resistance prevalence based on microbiology data.

Resistance Type	N Patients	Prevalence (%)
MRSA	9	7.1
MRCoNS	4	3.2
VRE	0	0.0
ESBL	5	4.0
3−ceph	4	3.2
CRE	0	0.0
ESBL−NF	1	0.8
CR−NF	2	1.6
Other MDR	11	8.7
**Overall resistance prevalence**	**33**	**26.0**

N is the number of patients reported to have received a microbiology-based treatment and prevalence (%) = 100 × (the number of patients reported to have received a microbiology-based treatment/total number of patients (N = 127) receiving a treatment with at least one antibacterial for systemic use (ATC J01) for a community-acquired or healthcare-associated infection). Overall resistance prevalence provides the total number of patients concerned, indicating that some patients were identified with more than one resistant organism with the overall resistance prevalence (%) at hospital level. **MRSA** = targeting methicillin-resistant *Staphylococcus aureus*; **MRCoNS** = targeting methicillin-resistant coagulase-negative staphylococci; **VRE** = targeting vancomycin-resistant enterococci; **ESBL** = targeting Enterobacteriales producing extended-spectrum beta-lactamase. **3-ceph** = targeting third-generation cephalosporin-resistant Enterobacteriales; Enterobacteriales and either the ESBL status is unknown or another resistance mechanism is present. **CRE** = targeting carbapenem-resistant Enterobacteriales; **ESBL-NF** = targeting ESBL-producing nonfermenter Gram-negative bacilli targeting nonfermenters (*Pseudomonas aeruginosa*, *Acinetobacter baumannii*, *Burkholderia* spp., *Stenotrophomonas maltophilia*) producing extended-spectrum beta-lactamase. **CR_NF** = targeting carbapenem-resistant nonfermenters (*Pseudomonas aeruginosa*, *Acinetobacter baumannii*, *Burkholderia* spp., *Stenotrophomonas maltophilia*). **Other MDRO** = targeting multidrug resistant organisms other than the ones listed above.

## Data Availability

Data available on request due to privacy/ethical restrictions.

## Supplementary Materials

The following global PPS are available online at www.global-pps.com.
